# Autonomic Regulation of Facial Temperature during Stress: A Cross-Mapping Analysis

**DOI:** 10.3390/s23146403

**Published:** 2023-07-14

**Authors:** Federica Gioia, Mimma Nardelli, Enzo Pasquale Scilingo, Alberto Greco

**Affiliations:** 1 Dipartimento di Ingegneria dell’Informazione, University of Pisa, 56122 Pisa, Italy; federica.gioia@phd.unipi.it (F.G.); mimma.nardelli@unipi.it (M.N.); enzo.scilingo@unipi.it (E.P.S.); 2 Research Center “E. Piaggio”, University of Pisa, Largo Lucio Lazzarino, 1, 56122 Pisa, Italy

**Keywords:** infrared thermography, cross-mapping, heart rate variability, electrodermal activity

## Abstract

Skin temperature reflects the Autonomic Nervous System (ANS)’s response to emotions and mental states and can be remotely measured using InfraRed Thermography. Understanding the physiological mechanisms that affect facial temperature is essential to improve the precision of emotional inference from thermal imaging. To achieve this aim, we recorded thermal images from 30 volunteers, at rest and under acute stress induced by the Stroop test, together with two autonomic correlates, i.e., heart rate variability and electrodermal activity, the former serving as a measure of cardiovascular dynamics, and the latter of the activity of the sweat glands. We used a Cross Mapping (CM) approach to quantify the nonlinear coupling of the temperature from four facial regions with the ANS correlates. CM reveals that facial temperature has a statistically significant correlation with the two autonomic time series, under both conditions, which was not evident in the linear domain. In particular, compared to the other regions, the nose shows a significantly higher link to the electrodermal activity in both conditions, and to the heart rate variability under stress. Moreover, the cardiovascular activity seems to be primarily responsible for the well-known decrease in nose temperature, and its coupling with the thermal signals significantly varies with gender.

## 1. Introduction

InfraRed Thermal Imaging (IRT) is taking hold in many sectors as a powerful contactless monitoring tool. Thermal cameras convert the IR energy emitted by any object at a higher temperature than absolute zero to a thermal image, also called a thermogram. Hence, through IRT we can visualize, measure and monitor an entire scene in thermal. In particular, in the psychophysiological field, thermal imaging has captured the attention of researchers since it could potentially reveal the subjects’ emotional state [1].

Numerous studies have investigated the level of autonomic emotion specificity by examining the various trends in peripheral physiological reactions to a range of emotional stimuli [2,3,4]. However, researchers seek a non-intrusive and contactless monitoring device to replace the commonly used wearable systems, which rely on electrodes positioned on the skin of the subject and may include cables connecting them to the acquisition unit. In fact, these tools are likely to interfere with the subject, compromising the natural physiological responses under study.

The body’s thermal response to affective stimuli is likely a dynamic process influenced by various physiological and psychological factors. This complexity presents a challenge in the accurate interpretation of thermal imaging data in psychophysiological research. The autonomic nervous system (ANS), which regulates the visceral responses to a change in mental state, seems to be responsible for the thermal modulation of the skin. Indeed, ANS regulates sweat production and influences the vasomotor activity of the subcutaneous vessels and consequently the dynamics of the blood circulation which produces the temperature variation [5,6,7,8]. A higher blood flux would cause a rise in local skin temperature, in contrast to perspiration which would cause a drop in temperature, due to the production of cold sweat. Moreover, respiration, muscle tension, and metabolic processes may also play a role in thermal regulation [6,9,10].

Therefore, thermal imaging has the potential to provide valuable insights into the physiological mechanisms underlying psychological states and behaviours. However, there is limited knowledge about how the interplay of these phenomena leads to the final temperature. Modelling these processes could ease the evaluation of experimental results, leading to higher specificity and interpretability. To achieve this aim, it is important to consider the nonlinear nature of the vast majority of biological systems including the ANS processes [11]. In fact, the analysis of nonlinear dynamics of ANS correlates, such as heart rate variability (HRV) and electrodermal activity (EDA), has shed light on the regulation of the organism’s homeostasis by the ANS in the face of numerous internal and external stimuli [12,13,14]. Furthermore, previous studies have shown that many of the biological processes related to human thermoregulation also show chaotic behaviour [15,16,17].

Another important aspect to take into consideration when investigating thermoregulation is that thermal dynamics differ based on gender. In particular, differences in thermoregulation have been associated with subcutaneous fat content, menstrual cycles, and sweat evaporation efficiency [18,19]. According to Christensen et al., males have a higher average facial skin temperature than females due to differences in blood circulation in the skin or the higher basic metabolic rate in males [20].

In our study, we aim to provide insights into the ANS nonlinear processes underlying facial skin temperature regulation in response to emotional stimuli. Specifically, we analyze how cardiovascular and sweat gland dynamics, in terms of HRV and EDA, influence thermal modulation, focusing also on gender differences. Indeed, HRV dynamics reflects the interplay between the sympathetic and parasympathetic nervous systems, EDA is the result of the sympathetic regulation only, and together they could quantify the two ANS sub-system activities. To quantify the nonlinear correlations between IRT and both HRV and EDA time series, we used a cross-mapping approach under the assumption that two time series generated by the same underlying system should have comparable dynamical characteristics and behavioural patterns [21,22,23]. In fact, crossmapping provides a powerful tool for exploring the similarity between the two time series and determining whether they are produced by the same dynamical system. Particularly, we applied this analysis to thermal signals extracted from four facial regions recorded from healthy subjects during rest and under a stressful stimulus, to address the question both under parasympathetic and sympathetic nervous system predominance experimental conditions, respectively.

## 2. Materials and Methods

### 2.1. Study Population

We recruited 30 healthy Caucasian volunteers (20 females, age = 26.6 ± 3.6 years) to participate in this study. We specifically chose individuals whose body mass index (BMI) was in the healthy range and who had no history of mental illnesses. While the BMI was controlled to reduce any potential effects of fat tissue on the thermal behavior of the skin, mental problems can possibly affect a variety of physiological and psychological aspects. The experiments were approved by the “Bioethics Committee of the University of Pisa” (n. 15/2019), and each volunteer signed the informed consent. All subjects were asked not to drink coffee or smoke on the same day of the experiment to avoid the potential vasomotor effects of these substances. Finally, the day of the experiment, subjects were not exposed to extrinsic factors affecting skin temperature (e.g., massages, epilation or physical activity).

### 2.2. Experimental Protocol

**Figure 1:**
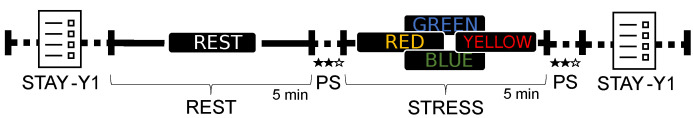
Protocol Timeline. The dashed line indicates non-timed tasks. PS—Perceived Stress self-assessment.

Before the experiment, the subject sat quietly in the experimental room for at least 10 min to acclimatize to the environmental setting. The experimental timeline is shown in Figure 1. The experiment consisted of a 5-min resting session followed by a 5-min stressor task.

The instructions were presented on a laptop as the experimenter left the room to avoid interactions with the participant and equalize conditions for all the volunteers. During the resting session, the subject was required to rest with eyes open. Then, the affective elicitation was performed using a computerized-paced Stroop Test, aimed to induce mental stress [24]. This is a well-known task that requires the resolution of incongruous color/semantic-meaning stimuli. Here, the subject was required to press a button corresponding to the tint of the word presented in the middle of the screen. The stimuli were paced every 2 s. At the top of the screen, a counter kept track of the number of successes, as a motivational stressor. At the start and the end of the experiment, the subject was required to fill in the State-Trait Anxiety Inventory (STAY-Y1), a psychological inventory consisting of 20 self-report items on a four-point Likert scale to measure state anxiety. Before and after the stressor, a self-assessed perceived stress (PS) level was reported on a Likert scale from 0 (not at all) to 10 (very stressed), and it served as a reference for our analysis.

Environmental conditions were kept constant during each experimental session using a thermostat. In particular, room atmospheric temperature and humidity ranged around 24–26 ${}^{{}^{\circ}}$C and 40–60%, respectively. Moreover, within the same experimental session, the temperature change was never over 1 ${}^{{}^{\circ}}$C and humidity variation was a maximum of 5%. All recordings were obtained in the same room, and the participant was never exposed to any direct light sources or ventilation. A chinrest guaranteed that the camera was always perpendicular to the face of the subject and at a fixed distance of 1 m. The camera’s emissivity was set to 0.98, the recommended value for a human body surface. Twenty minutes before the start of the recording, the camera was turned on to allow for sensor stabilization. All the points of the checklist described in [25] were addressed to guarantee reliable skin temperature recordings with infrared thermography.

### 2.3. Equipment and Data Acquisition

Facial thermograms were acquired using a FLIR T640 thermal camera with a 24.6 mm lens, 640 × 480 pixels, NETD < 0.04 mK @ +30${}^{{}^{\circ}}$ and spectral range of 7.8–14 μm (Long-wave-infrared, LWIR). The sampling frequency was 5 Hz. In addition, RGB images in the visible spectrum were recorded using a Logitech HD WebCam C270. Physiological signals (i.e., electrodermal and cardiac activity) were acquired using a BIOPAC MP150 system, at a sampling rate of 250 Hz. Specifically, EDA electrodes were placed on the distal phalanx of the index and ring fingers of the non-dominant hand. Electrocardiogram (ECG) was recorded using three electrodes, respectively below the right and left clavicle, and on the lower left chest.

### 2.4. Data Pre-Processing

**Figure 2:**
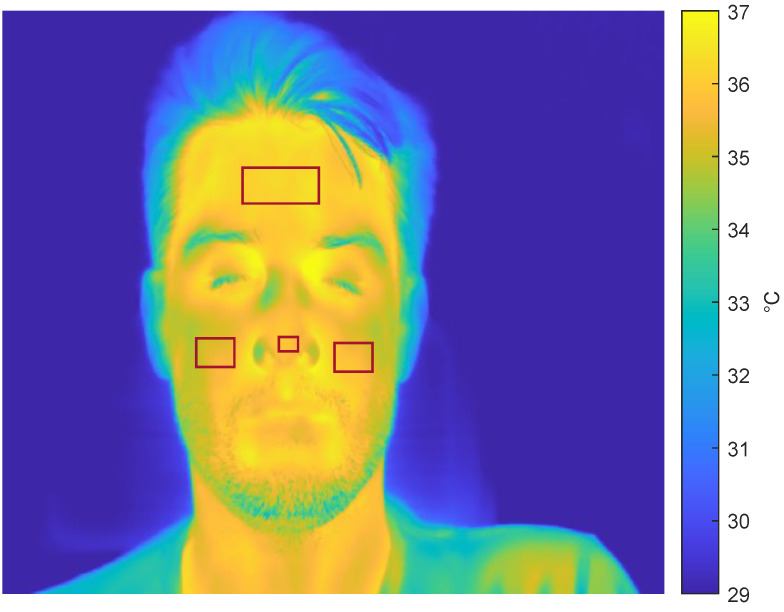
Facial ROIs location.

We used thermal imaging to monitor the temperature of several regions of interest (ROIs) of the face (i.e., nose, forehead, and cheeks), as shown in Figure 2 [26,27].

Firstly, IR images were co-registered using a motion correction framework based on optical flow [5]. We used RGB images to localize the position of the ROIs in the first frame of each IR recording. To accomplish this aim, RGB and IR videos were synchronized in time. In addition, the first frames from the two techniques were spatially aligned using fiducial marker-based registration with an affine-2D transformation matrix. Then, we identified 68 facial landmarks in the RGB domain using the Yuval Nirkin algorithm [28]. Based on the position of these landmarks, the centers of the ROIs were automatically detected. The algorithm to locate the ROIs was developed in our previous study [27], and it is based on the intersection of lines connecting specific landmarks. In order to adapt the ROIs to each face shape and size, the dimension of each ROI was calculated to be proportional to the number of face pixels. Table 1 presents the mean and standard deviation of the number of pixels per ROI across all subjects in our study.

Accordingly, the face was separated from the background using a landmark-intensity-based segmentation to count the number of face pixels. Finally, the coordinates of the ROIs in the first RGB frame were referred to the co-registered IR images, in order to obtain the thermal time-series of each region. Each point of the thermal time-series is the median temperature value of the pixels within a ROI of a specific frame. The temperature was quite uniform within each ROI, as indicated by the average Median Absolute Deviation (MAD) of the temperature within each ROI, across frames and subjects, reported in Table 2. As a result, we focused on the median value because it provides a reliable approximation of the temperature inside the ROI and is unaffected by artifacts.

For the analysis, we selected 150-second-long segments of the thermal time-series. In particular, the last half of the resting session was adopted as a baseline, while the first half of the stressor task was chosen as the stimulation phase. On the one hand, this choice insured that during the baseline the subject had completely recovered from the distress caused by the setup process. On the other, it minimized the habituation effect on the stimulation phase. Hereinafter, we will refer to these segments as *Rest* and *Stress*, respectively. Finally, we extracted the median temperature during *Rest* and *Stress*.

ECG signals were processed using Kubios HRV software [29] to obtain the Heart Rate Variability (HRV) series. The interbeat (RR) signals were obtained using the Pan–Tompkins algorithm. In particular, a pre-processing phase was followed by decision rules to detect the QRS complex. Firstly, the ECG was bandpass filtered to eliminate power line noise and baseline drift. Then, the data samples were squared to highlight peaks, and moving average filtered to soften nearby peaks. The amplitude threshold and comparison to the predicted value between neighbouring R-waves are part of the decision rules. Every time a new R-wave was successfully identified, both of these rules were adaptively modified. The artifacts present in some of the signals were corrected using the threshold-based artifact correction algorithm proposed by the same software. Essentially, artefacts are corrected by comparing each RR interval value to a local average interval, obtained by median filtering the time series. If an RR interval differs from the local average more than a threshold, the interval is identified as an artifact and is marked for correction. The lowest threshold (0.45 s) was chosen for artifact detection. A cubic spline interpolation is used to replace the identified artefacts with interpolated values to make the fix. Afterwards, to obtain uniformly sampled signals, the RR series were interpolated using a piecewise cubic interpolation at 5 Hz, matching the IR sampling frequency. For the analysis, we selected the 150-s segments of HRV time-series corresponding to the ones selected for the thermal signal, for analogous reasons. For each HRV segment, we extracted several features related to the cardiovascular activity, as reported in Table 3.

The EDA signal reflects the activity of the sweat glands, which causes variations in the electrical properties of the skin. Sweat secretion in response to emotional stimuli is regulated by the sympathetic branch of the ANS. Therefore, EDA is a well-established signal to monitor the activity of the SNS in relation to changes in mental state. The EDA signal has a tonic component, henceforth referred to as EDAtonic, whose spectrum is below 0.05 Hz. The EDAtonic reflects the sympathetic tone and is related to changes in the overall state of the subject. Conversely, short-term and event-related responses are reflected in the phasic component. Here, we used the cvxEDA algorithm to extract these two components from the raw EDA signal. This algorithm performs a model-based signal decomposition exploiting Bayesian statistics, convex optimization, and sparsity, without the need for pre- and post-processing steps [30]. Afterwards, the tonic and phasic components were downsampled to 5 Hz, to obtain the same time points as the thermal and HRV series. In addition, we selected the 150-s segments *Rest* and *Stress*.

Of note, two female subjects were excluded from the analysis due to the presence of artifacts. Finally, from each segment of both the tonic and phasic components, we extracted well-known features related to the SNS activity, as listed in Table 4.

### 2.5. Cross-Mapping

In this study, we applied the cross-mapping procedure to investigate the nature of the facial thermal dynamics of healthy subjects in response to an external emotional stimulus. Based on the previous literature, we hypothesize that the main phenomena underlying a change in skin temperature are related to both sweat glands and cardiovascular dynamics [5,31,32]. Since these two phenomena are directly correlated to HRV and EDA, we applied the cross-mapping procedure to each couple of time series composed of a ANS correlate (i.e., HRV, EDAtonic) and a thermal signal, respectively denoted as X = (X${}_{1}$, X${}_{2}$, …, X${}_{n}$) and Y = (Y${}_{1}$, Y${}_{2}$, …, Y${}_{n}$). Of note, only the tonic component of the EDA signal was selected for this analysis, as it has comparable dynamics with the thermal signals. Specifically, we estimated the thermal signals of each ROI from the reconstructed manifolds of each physiological signal M${}_{X}$. Firstly, we used Takens’ Time-Delay Embedding Theorem to reconstruct the attractor of each time-series [33]. All the time series were simultaneously recorded and uniformly resampled at a frequency of 5 Hz. According to Takens’ theorem, we can trace the trajectory of a dynamic system in the phase space having only one time series and two parameters: the dimension of the phase space *m* and the time delay τ. Both of these parameters are unknown but can be estimated using well-known methods. Here, the value of τ was selected as the lag corresponding to the first minimum of the automutual information (MI) function of the time series used to reconstruct the phase space, ensuring statistical independence between the values, in both linear and nonlinear terms [34]. The embedding dimension *m* was selected using the False Nearest Neighbors algorithm (FNN), which is the most popular tool for the selection of the minimal embedding dimension [35]. This method is based on the assumption that if *m* is too small to unfold the attractor, points that are far from each other can appear as neighbours simply because the actual geometry of the attractor has been projected down onto a smaller space. These points are called false neighbours as they will no longer be neighbours when increasing the embedding dimension. Therefore, the algorithm iteratively increases the embedding dimension and at each step calculates the number of nearest neighbours. The final *m* is chosen as the dimension for which the change in the number of false neighbours compared to the previous iteration is below a threshold. Here, we used the algorithms proposed in [36,37] to select the values of τ and *m*, respectively.

The reconstructed attractors, are referred to as shadow manifolds in [21], and they are approximations of the true attractor. Indeed, the shadow manifolds of each variable is diffeomorphic from the original manifold. Takens’ theorem demonstrates that when two different variables are representations of the same dynamical system, their shadow manifolds are diffeomorphic to each other. Hence, if both X and Y dynamics are driven by the same underlying dynamical system, their manifolds are diffeomorphic and we can obtain the estimate of Y from the manifold M${}_{X}$, i.e., $\hat{Y}$|M${}_{X}$. In this study, we obtained $\hat{Y}$|M${}_{X}$ following the procedure described in [38]. More specifically, since M${}_{X}$ is diffeomorphic to M${}_{Y}$, points that are nearby in M${}_{X}$ correspond to points that are also nearby in M${}_{Y}$. Therefore, we identified the *m*+1 nearest-neighbours in M${}_{X}$ over time, and their corresponding points in M${}_{Y}$. The number of points depends on the maximum value of embedding dimension *m* between the two time series. The latter were used to map a region of *m*+1 points around Y${}_{t}$. The estimate of each point of Y starting from the reconstructed manifold of X is found as follows:

$${\hat{Y}}_{t}|M_{X}=\sum_{i=1}^{m+1}\omega_{i}Y_{t_{i}}$$

where $\omega_{i}$ are the weights obtained as the Euclidean distances between X${}_{t}$ and the nearest-neighbours points (||·|| indicates the Euclidean distance in $R^{m}$):

$$\omega_{i}=\frac{u_{i}}{\sum_{j=1}^{m+1}u_{j}}$$

$$u_{i}=exp\left[-\frac{\left|\right|X_{t}-X_{t_{i}}\left|\right|}{\left|\right|X_{t}-X_{t_{1}}\left|\right|}\right]$$

**Figure 3:**
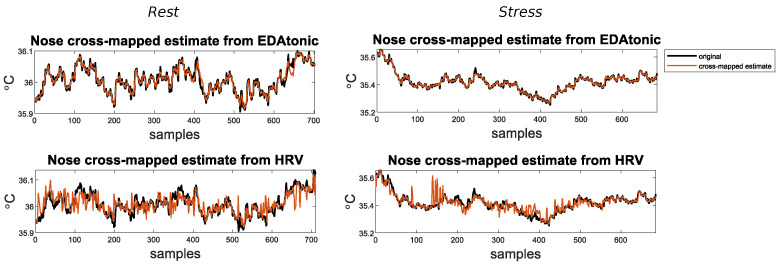
Cross-mapped estimates of the thermal signal of the nose (**Nose**) from each physiological time-series (i.e., **EDAtonic**, **HRV**), for a single subject in the two conditions *Rest* (Left), and *Stress* (Right). Each cross-mapped estimate is superimposed on the original time series.

In Figure 3, we show, for a single subject in the two sessions, the estimates of the thermal signal of the nose from each physiological time series.

Finally, we computed the Spearman correlation coefficients between each original thermal signal and the corresponding estimate obtained using cross-mapping, for each subject during each condition, and we will refer to them as $\rho_{Y\hat{Y}}$.

### 2.6. Statistical Analysis

We performed statistical analysis to explore at the group level the ANS correlates and the psychometric variation between the two sessions, *Rest* and *Stress*. The non-parametric Wilcoxon sign-rank test was used for each thermal, EDA and HRV feature between *Rest* and *Stress* (α = 0.05). Similarly, the same test was used to assess variations between the PS level and the STAY-Y1 total score reported before and after the *Stress*. We controlled false discovery rate through the Benjamini, Krieger, and Yekutieli (FDR-BKY) correction for multiple hypothesis testing [39].

We assessed the significance level of each correlation coefficient $\rho_{\hat{Y}Y}$ using a non-parametric permutation test with 1000 repetitions. The original thermal signal was divided into blocks of length *L*, equal to τ, which were randomly shuffled at each iteration to generate the null distribution. Similarly, we computed the Spearman correlation coefficients between the original thermal signals and each physiological signal, $\rho_{XY}$, and their significance levels were assessed using the same permutation test.

For each physiological signal used to obtain the cross-mapped estimate of a thermal signal, we performed Wilcoxon sign-rank test with Bonferroni correction among the four different facial ROIs to test for variations in the correlation coefficient $\rho_{\hat{Y}Y}$.

Finally, we performed a split-plot analysis of variance (SPANOVA), to statistically compare group-wise means between $\rho_{\hat{Y}Y}$ values obtained after the application of the cross-mapping procedure to each couple of time-series X, Y. Specifically, this statistical analysis consisted of testing for significant differences in the $\rho_{\hat{Y}Y}$ values between female and male participants, while subjecting participants to repeated measures. Thus, there is one between-subjects variable (Female vs. Male) and one within-subjects variable (*Rest* vs. *Stress*). This analysis was performed in RStudio, using the package rstatix.

## 3. Results

The statistical analysis on both psychometric tests indicated that subjects reported being more stressed after the stressor compared to before. In particular, the Wilcoxon test on the PS levels reported a *p*-value equal to 0.01, and the *p*-value for the STAY-Y1 total scores was equal to 0.03.

**Figure 4:**
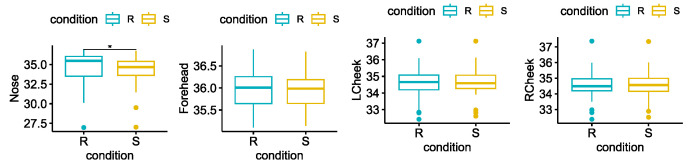
Statistical comparison of the median temperature, for each facial region of interest. Significant differences between *Rest* = R and *Stress* = S after the Wilcoxon signed rank test with FDR-BKY correction are highlighted with an asterisk (* = *p* < 0.05).

Among the facial regions, the nose was the only ROI to change significantly in the two conditions. In particular, the median temperature of the nose decreased significantly during *Stress*, as shown in Figure 4.

**Figure 5:**
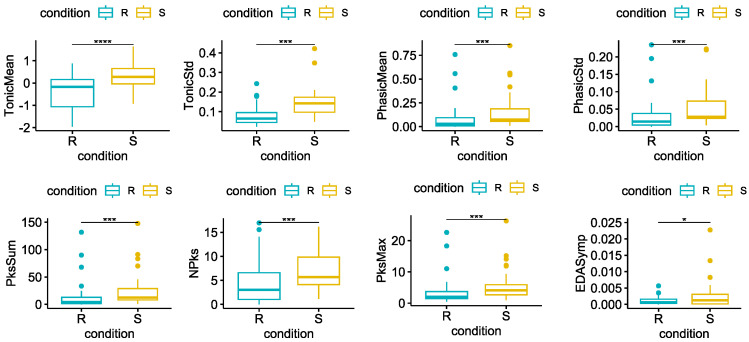
Statistical comparison for each of the EDA features extracted. Significant differences between *Rest* = R and *Stress* = S after the Wilcoxon signed rank test with FDR-BKY correction are highlighted with an asterisk (* = *p* < 0.05; *** = *p* < 0.001, **** = *p* < 0.0001).

Regarding the ANS correlates data analysis, except for the *LF/HF ratio*, all the features extracted from the EDA and HRV confirmed a significant variation between *Rest* and *Stress*. Specifically, all the EDA features significantly increased during the stimulation, as shown in Figure 5.

**Figure 6:**
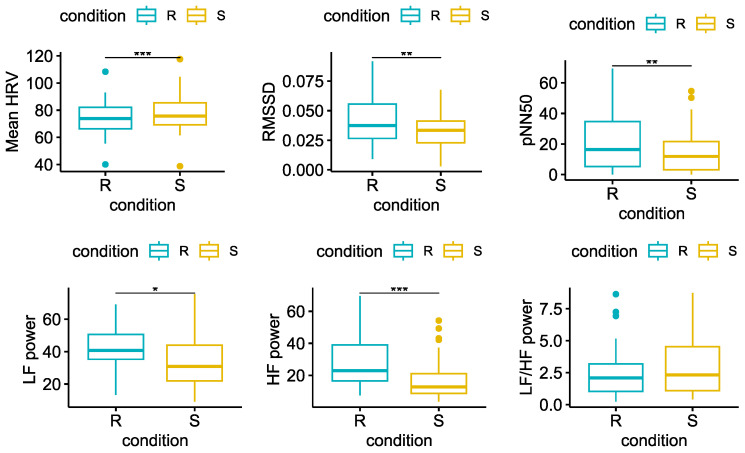
Statistical comparison for each of the HRV features extracted. Significant differences between *Rest* = R and *Stress* = S after the Wilcoxon signed rank test with FDR-BKY correction are highlighted with an asterisk (* = *p* < 0.05; ** = *p* < 0.01; *** = *p* < 0.001).

Likewise, the mean of HRV increased significantly during the *Stress*. Conversely, the remaining HRV features, except the *LF/HF ratio*, decreased significantly compared to the *Rest*, as shown in Figure 6.

Regarding the correlation coefficients $\rho_{XY}$, over 50% of the subjects showed a significant correlation between the time series of the tonic component of the EDA signal and each thermal signal. The correlation between the HRV time series and the thermal signals was significant for less than 50% of the subjects. However, the median $\rho_{XY}$ absolute values were always lower than 0.26, in both conditions. On the contrary, the *p*-values obtained for the correlation coefficients $\rho_{\hat{Y}Y}$ were lower than 0.05, and the median $\rho_{\hat{Y}Y}$ absolute values were always higher than 0.62, as shown in Table 5.

**Figure 7:**
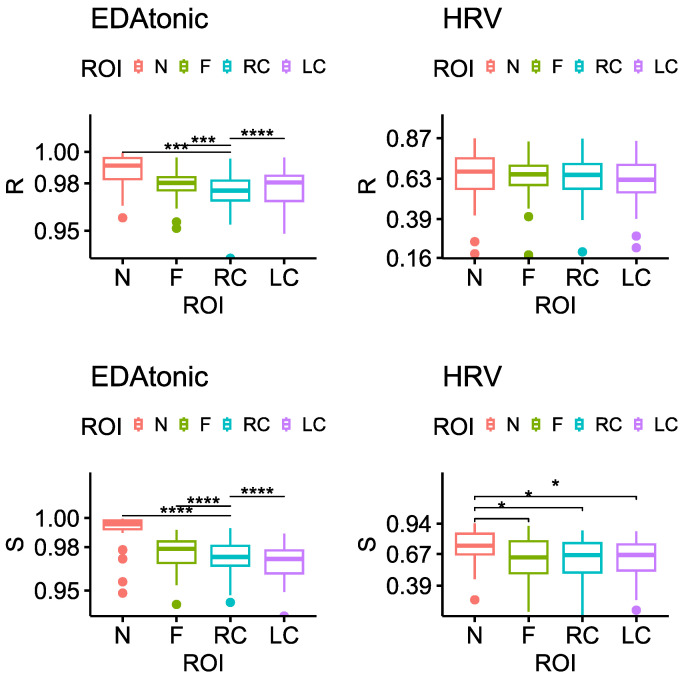
Statistical comparison of the $\rho_{\hat{Y}Y}$ obtained as the correlation between the $\hat{Y}$ estimated from the same physiological signal and Y corresponding to each facial ROI (Nose = N; Forehead = F; RC = Right Cheek; LC = Left Cheek), during both conditions (R = *Rest* and S = *Stress*). Significant differences between facial ROIs after the Wilcoxon signed rank test with Bonferroni correction are highlighted with an asterisk (* = *p* < 0.05; *** = *p* < 0.001; **** = *p* < 0.0001).

The results of the statistical analysis performed on the $\rho_{\hat{Y}Y}$ between facial ROIs are shown in Figure 7. The $\rho_{\hat{Y}Y}$ related to the thermal signal of the Nose region significantly differed from the others (i.e., Forehead, RCheek, LCheek) when estimated from the *EDAtonic* during both *Rest* and *Stress*, and when estimated from the *HRV* during *Stress*. In particular, the $\rho_{\hat{Y}Y}$ between the original thermal signal of the Nose and the same thermal signal estimated from each physiological signal, was always higher compared to the corresponding correlation coefficient for the other regions.

**Figure 8:**
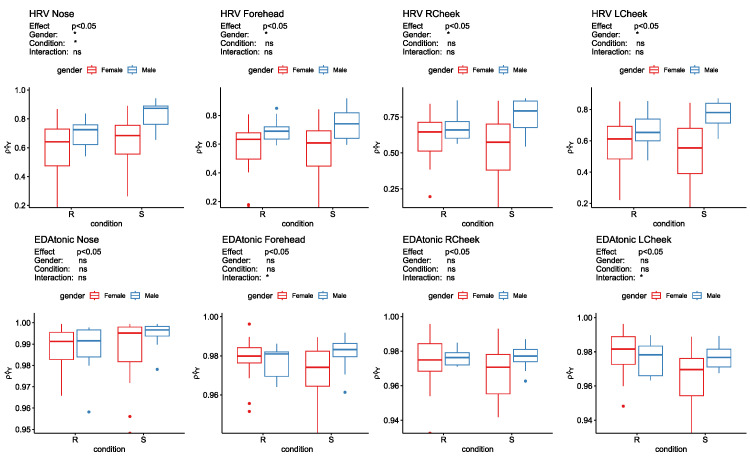
Split-Plot Anova results for each correlation coefficient $\rho_{\hat{Y}Y}$ obtained as the correlation between the time-series $\hat{Y}$ estimated from each physiological signal (i.e., HRV, EDAtonic) and Y corresponding to each facial ROI (Nose, Forehead, Right Cheek, Left Cheek); ns = non-significant; * = significant; Rest = R and Stress = S.

Finally, the results of the SPANOVA on the $\rho_{\hat{Y}Y}$ values are shown in Figure 8. As for the “between subjects” effect, the null hypothesis was that females and males have equal $\rho_{\hat{Y}Y}$ values, on average. This hypothesis was always rejected at the alpha level equal to 0.05 when comparing the $\rho_{\hat{Y}Y}$ values obtained after applying the cross-mapping approach to the HRV time series and each thermal signal.

The main effect for the condition, whose levels are *Rest* and *Stress*, was significant only for the $\rho_{\hat{Y}Y}$ values obtained after applying the cross-mapping approach to the HRV time series and the thermal signal of the nose (*p*-value = 0.03). In particular, the $\rho_{\hat{Y}Y}$ values increased during *Stress*. In this case, the interaction between gender and condition was not significant, indicating that the effect of the condition variable did not differ significantly between males and females. Finally, no significant differences were found between condition or gender in the correlation coefficients obtained after applying the cross-mapping approach to the EDA time series and the thermal signals from any ROI. However, the interaction term for the $\rho_{\hat{Y}Y}$, where $\hat{Y}$ is the cross-mapped estimate of the Forehead and Left Cheek’s temperature signal from the EDAtonic time series, was significant (*p*-value equal to 0.031 and 0.034, respectively). Hence, the effect of the condition variable on the correlation coefficient $\rho_{\hat{Y}Y}$ varied depending on the level of the gender variable. Specifically, for the Forehead, the effect of the condition variable on the correlation coefficient switched direction for males and females, resulting in a crossover pattern, as visible in Figure 8. In fact, $\rho_{\hat{Y}Y}$ increases in men during *Stress* compared to *Rest*, but it decreases in women.

## 4. Discussion

This study explores the physiological phenomena driving facial temperature modulation, under both parasympathetic and sympathetic dominance. Many studies have associated the temperature drop in the nose to a sympathetically driven vasoconstriction of the subcutaneous vessel, in response to a stressful stimulus [31,32,40,41]. Similar to this, perspiration brought on by stress or high arousal stimuli was linked to a temperature drop in the upperlip region with a specific dotted pattern [42,43]. Here, we wanted to give a foundation to the hypotheses that have prevailed in the interpretation of the results obtained in thermographic studies in psychophysiology. In particular, we performed a nonlinear analysis based on chaos theory, to investigate the imprint that the cardiovascular and sudomotor activity have on the temperature extracted from different regions of the face. Moreover, we quantified the influence that these two systems have on skin temperature, in two different conditions, namely resting state and stress. In addition, we explored potential gender differences in the physiology behind thermal modulation.

First of all, we confirmed the effectiveness of the protocol in inducing stress during the stimulation by comparing the psychometric scores reported before and after the stressor. Objective physiological measures gave us further evidence of the increased arousal during the stressor. Although the features we extracted are naturally standardized in the physiological ranges, outliers are frequently present in studies of physiological aspects due to the high intrinsic subject variability. To limit the potential impact of these outliers on our results, we have used non-parametric tests that are less influenced by extreme values. All the features extracted from the EDA signal, increased significantly during *Stress*. Similarly, all the HRV features except one (the LF/HF ratio), both in the time and frequency domain, varied significantly in the expected direction between the two conditions. Finally, the median temperature of the nose decreased during *Stress*, as widely described in the literature [26].

Nonlinear analysis of the thermal and HRV time series revealed a strong relation between the thermal and cardiovascular dynamics that was not obvious in the linear domain. Indeed, the linear correlation between the original HRV and thermal series was significant in the majority of the subjects only for the nose region during *Stress*, and the correlation values $\rho_{XY}$ reported a median value lower than 0.15. On the other hand, the correlation values between the thermal signals and their estimated version obtained using the cross-mapping approach with the HRV time series were significant for all the subjects during both *Rest* and *Stress*, with a median value higher than 0.7. Specifically, the nose region presented the highest median correlation value, that increased during *Stress* compared to *Rest*.

Conversely, the relation between the thermal and electrodermal dynamics was already visible in the linear domain, as the linear correlation was significant in the majority of the subjects for all the facial regions and in both conditions. However, median correlation values $\rho_{XY}$ were lower than 0.25. In this case, the correlation values between the thermal signals and their estimated version obtained using the cross-mapping approach with the time series of tonic component of the EDA were always significant and their median value was close to 1. This result suggests that the thermal signals from the four selected regions and the tonic electrodermal component describe the dynamics of the same system, thus perspiration seems to play an important role in the facial thermal modulation, possibly higher than the vascular activity.

The nonlinear correlation values quantify how the knowledge of the shadow manifold obtained from the time series of the physiological time series can be used to estimate values of the thermal time series. Hence, we used the correlation value $\rho_{\hat{Y}Y}$ as an index of the ‘imprint’ of the EDA and HRV time series on the thermal signals of the most commonly selected regions of the face. A comparison of the nonlinear correlation values obtained for the different facial regions reveals that, although the correlation coefficient is very high for all the regions, the nose has a significantly higher influence of the electrodermal activity compared to the other face areas, during both conditions. On the other hand, the influence of cardiovascular activity seems to be similar in all the regions during *Rest*, while it is significantly higher in the nose during *Stress*. This different behaviour of EDA- and HRV-estimated thermal signals between rest and stress is confirmed by the SPANOVA comparison among conditions and genders. In fact, the comparison between conditions and genders was never significant when considering the EDA-estimated thermal signal of the nose, as well as of the other regions. In fact, the EDA appears to have a significant impact on all facial temperatures, at a similar level in both conditions and without obvious gender variations. The imprint of the HRV on the variation of the thermal signal from the four ROIs varies significantly between the rest and stress. In particular, the contribution of the HRV is on average higher during stress in the nose and forehead. Hence, we can speculate that the well-known decrease in temperature of the nose tip during stress is mostly due to the sympathovagal-driven vasoconstriction of the subcutaneous vessel. The median temperature of the forehead did not change significantly during stress, as shown from the first analysis by means of the Wilcoxon signed-rank test. Thus, we cannot associate the increase in the $\rho_{\hat{Y}Y}$ with a particular physiological phenomenon with the same confidence. Our findings indicate a gender-dependent variation between stress and rest states. Specifically, in contrast to their male counterparts, female subjects demonstrated a significant decrease in correlation values under stress conditions, albeit the average correlation values remained high. This observed difference may potentially be indicative of inherent biological gender disparities within these regions of interest. However, it is worthwhile noting that these are preliminary findings and further comprehensive investigations are required to draw definitive conclusions.

A limitation of this study is gender imbalance. The disparity between males and females in our study population could have, in fact, hidden some other results that would have led to a better interpretation. In general, the sample size we had available was limited. Hence, future work will expand the study to a larger cohort of subjects to confirm the findings and strengthen the conclusions drawn. Moreover, we intent to compare thermal modulation in different populations. Indeed, the sympathetic and parasympathetic nervous systems’ responses to emotional stress can vary depending not only on gender, but also age or clinical conditions. Hence, comparing thermal modulation in various groups, can help to comprehend the underlying mechanisms and potentially lead to customised therapies.

## 5. Conclusions

In conclusion, our study presented a novel approach to the study of the nonlinear nature of thermal modulation. We have researched and, in a preliminary manner, shown the effect of both the sympathetic or parasympathetic autonomic subsystems on the facial temperature variation in response to emotional stress. Future work will explore the nonlinear coupling between the autonomic nervous system and the temperature of a broader range of facial regions, ideally encompassing the entire face. Advanced approaches like finite element methods may be employed to complete this thorough investigation. Understanding the mechanisms underlying these temperature variations may make it possible to create more precise techniques for stress detection and stress management, lie detection, and the prediction of underlying psychiatric conditions. Finally, the imprint of autonomic correlates in the thermal signal, whose existence we have demonstrated, paves the way for future research aimed at extracting information specifically related to HRV and EDA directly from the thermal signal.

## Figures and Tables

**Table 1:** Number of Pixels per ROI (mean ± standard deviation).

Nose	Forehead	LCheek	RCheek
275.1 ± 37.5	2540.2 ± 346.4	1288 ± 174.4	1288 ± 174.4

**Table 2:** Average Temperature within each ROI (Median ± MAD).

Nose	Forehead	LCheek	RCheek
34.23 ± 0.19	35.98 ± 0.13	34.38 ± 0.39	34.54 ± 0.4

**Table 3:** Features extracted from the HRV.

Feature	Description
*meanHRV*	Mean value of HRV
*stdHRV*	Standard deviation of HRV
*RMSSD*	Root mean square of successive RR interval differences
*pNN50*	Percentage of successive RR intervals that differ by more than 50 ms
*LF*	Percentage of the total power in the low-frequency (0.04–0.15 Hz)
*HF*	Percentage of the total power in the high-frequency (0.15–0.40 Hz)
*LF/HF ratio*	Ratio of LF to HF power

**Table 4:** Features extracted from the tonic and phasic components.

Feature	Description
*TonicMean*	Mean value of the tonic component *
*TonicStd*	Standard deviation of the tonic component *
*PhasicMean*	Mean value of the phasic component **
*PhasicStd*	Standard deviation of the phasic component **
*PksMax*	Maximum peak **
*NPks*	Number of peaks **
*PksSum*	Sum of peaks **
*EDASymp*	Power of the skin conductance spectrum in the range of 0.04–0.25 Hz

*: value averaged within non-overlapped 30-second-long time windows. **: value averaged within non-overlapped 5-second-long time windows.

**Table 5:** Percentage of significant correlation coefficients $\rho_{XY}$ and $\rho_{\hat{Y}Y}$ (*p* < 0.05) and median ρ value.

X										
			EDAtonic				HRV			
			REST		STRESS		REST		STRESS	
			%	ρ	%	ρ	%	ρ	%	ρ
Y	Nose	$\rho_{XY}$	75	−0.26	85.71	0.13	17.86	−0.02	60.71	−0.17
		$\rho_{\hat{Y}Y}$	100	0.99	100	0.99	100	0.67	100	0.74
	Forehead	$\rho_{XY}$	60.71	−0.02	57.14	0.02	7.14	0.02	39.29	0.03
		$\rho_{\hat{Y}Y}$	100	0.98	100 0.98	100	0.66	100	0.64	
	Rcheek	$\rho_{XY}$	53.57	−0.17	60.71	0.05	14.29	−0.01	28.57	−0.01
		$\rho_{\hat{Y}Y}$	100	0.98	100	0.97	100	0.65	100	0.66
	Lcheek	$\rho_{XY}$	71.43	−0.11	64.29	0.02	10.71	0.02	32.14	0.01
		$\rho_{\hat{Y}Y}$	100	0.98	100	0.97	100	0.62	100	0.66

## Data Availability

The data are not publicly available due to privacy restrictions.
